# Gait characteristics in idiopathic normal pressure hydrocephalus: A controlled study using an inertial sensor system

**DOI:** 10.1371/journal.pone.0317901

**Published:** 2025-02-26

**Authors:** Johanna Rydja, Petra Pohl, Andreas Eleftheriou, Fredrik Lundin

**Affiliations:** 1 Department of Activity and Health, and Department of Biomedical and Clinical Sciences, Linköping University, Linköping, Sweden; 2 Department of Health and Rehabilitation, Institute of Neuroscience and Physiology, Sahlgrenska Academy, University of Gothenburg, Göteborg, Sweden; 3 Department of Neurology, and Department of Biomedical and Clinical Sciences, Linköping University, Linköping, Sweden; IRCCS Medea: Istituto di Ricovero e Cura a Carattere Scientifico Eugenio Medea, ITALY

## Abstract

**Background:**

Gait disturbance is the most pronounced symptom in idiopathic normal pressure hydrocephalus (iNPH). Some gait parameters have been previously described, but an in-depth description of the gait pattern is lacking. The aim was to quantitatively evaluate gait in iNPH patients before and after shunt surgery and compare it to healthy individuals (HI), and correlate it with functional tests preoperatively.

**Methods:**

In total, 47 patients and 42 HI were included. The patients were assessed with the iNPH scale (total, gait and balance domain scores were analyzed), the timed up and go test (TUG) and an inertial sensor gait analysis system, RehaGait^®^, pre- and postoperatively. The HI were assessed with TUG and RehaGait^®^. Gait variables were: stride length, stride duration, velocity, cadence, variability, stance, swing, single support, double support, step height, hip, knee and ankle joint angles.

**Results:**

Compared to HI, the main differences in the gait variables were: decreased stride length (p < 0.01), velocity (p < 0.01), swing time (p < 0.01), single support (p < 0.01), hip flexion (p < 0.01), heel strike angle (p < 0.01) and toe-off angle (p < 0.01). Step height was normalized postoperatively; all other variables remained significantly worse than the HI. There were strong correlations between stride length, velocity, heel strike angle, and toe-off angle and the functional gait tests, but no correlations for any variable and the balance domain score.

**Conclusions:**

The patients walked with reduced hip flexion, heel strike angle and toe-off angle, and had shorter strides, decreased velocity, and increased time for swing and single support, compared to HI. Step height was the only gait variable normalized after shunt surgery. Ankle joint kinematics correlated strongly with the results in functional gait tests. More research is warranted about how gait speed affects other gait variables in iNPH.

**Trial registration:**

ClinicalTrials.gov NCT04795089

## Introduction

Idiopathic normal pressure hydrocephalus (iNPH) is a common condition among the elderly, caused by alterations in the cerebrospinal fluid circulation. Enlarged cerebral ventricles and typical radiological features, combined with normal intracranial pressure, are crucial for the diagnosis. INPH is treated with a surgical placement of a shunt to drain the excess fluid [1,2]. According to a systematic review from 2022, the prevalence of iNPH was reported at approximately 400/100,000 inhabitants and the incidence of surgery was only 1.7/100,000 [3]. Gait disturbance is the primary symptom, along with cognitive decline and impaired bladder control [4]. Even postural instability has been suggested as a characteristic symptom in iNPH, and impaired postural control is part of the gait disturbance [5–8].

The gait impairment in iNPH has been described in terms of typical signs and gait apraxia, where the patients have difficulty moving their legs rapidly in the gait situation but can move them, for example, while lying in bed [9]. The American-European guideline from 2005 emphasizes that two of the following gait characteristics should be present for a diagnosis: decreased step height, step length, and cadence; increased trunk sway during walking; widened standing base; toes turned outward on walking; turning requiring three or more steps for 180 degrees; and/or impaired walking balance, as evidenced by two or more corrections out of eight steps on tandem gait testing [1]. In the Japanese guidelines for management of iNPH (2021), three gait characteristics are highlighted: small-step gait, magnetic gait, and broad-based gait, and the gait is characterized as unstable and slow. The gait pattern is described as duck-footed with fluctuating stride length. When turning, the steps are small and unstable and freezing of gait may be present [10].

The typical iNPH gait signs have been questioned, and in clinical practice, a variation of gait disturbances is observed. In research, the gait characteristics are scarcely quantitatively described in relation to healthy individuals (HI) of the corresponding age. Over the years, only a few larger studies have utilized computerized objective analysis systems to evaluate gait characteristics. A frequently used system is a pressure-sensitive carpet that analyzes spatial and temporal gait parameters for a short distance [11–14]. Inertial sensor systems with sensors attached to the patient’s body to quantify gait variables have been used in a few studies [15–17]. The advantage of body-worn sensors is that they allow the patient to walk freely across the environment for a longer distance. The method also entails a more extensive analysis of the joint kinematics.

Some gait characteristics in iNPH have been established through previous research, such as impaired gait speed and cadence [11–14,16], short stride length and increased step width [11–14]. Stride length ≤1.02 m and velocity ≤ 0.83 m/s have been suggested as thresholds to discriminate between iNPH and HI [14]. However, for other parameters, clinical descriptions are often used without quantification. Scorings of gait disturbances according to typical iNPH gait signs are an essential part of the diagnostic process, and grading scales, such as the iNPH grading scale [18] or part of the iNPH scale [19], are used in the clinical evaluation. Gait and balance performances are also assessed using functional tests, including the 10-meter walk test (10MWT) [20] and Timed Up and Go test (TUG) [21]. The expression ”typical gait disturbance” in iNPH is often used, nevertheless the details about the gait are still unclear. The gait pattern in iNPH needs a more in-depth description, and the relation between gait variables and functional tests and scales have to be clarified.

The primary aim of this study was to explore the gait pattern in iNPH and quantitatively evaluate gait in patients with iNPH before and after shunt surgery and compare it to HI. Another aim was to evaluate the gait variables in patients with iNPH in relation to motor and balance tests used in the clinical evaluation.

## Materials and methods

The study was conducted in accordance with the declaration of Helsinski and approved by the Swedish Ethical Review Authority with approval number: 2020-00719. All patients and HI received written and verbal information and provided written consent for the study.

### Study population

This was a prospective study with a consecutive inclusion between October 15, 2020 and February 4, 2022 from the Hydrocephalus center at Linköping University hospital in Sweden. The patients were included in accordance with the inclusion criteria: iNPH diagnosis according to the American-European guidelines [1] and having undergone a shunt insertion. Patients were excluded if they were not able to walk 20 meters without a walking aid. Another reason for exclusion was inability to participate due to severe cognitive decline. The convenient sample of HI was recruited through an advertisement in the local newspaper and through recruitment among relatives and friends. The HI were examined by a neurologist (AE), had a cranial MRI, and were included if they were at least 60 years of age and had no signs of dementia, no notable gait disturbance, or any serious disease. The HI were included between February 24, 2022 and June 21, 2022.

**Fig 1:**
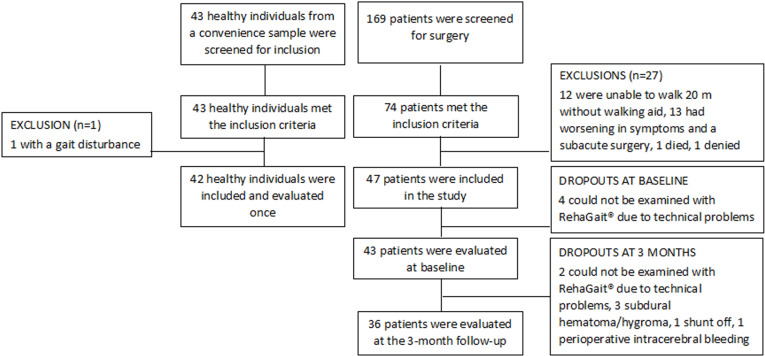
Flow chart of included patients and dropouts.

A total of 169 patients were screened for surgery during the inclusion period, and 74 patients met the inclusion criteria. Twenty-seven patients were excluded due to reasons explored in the figure (Fig 1). Four patients could not be evaluated due to technical problems with the gait analysis equipment, leaving 43 patients who were examined at baseline. At the three-month follow-up, there were seven dropouts (two due to technical problems, three due to subdural hygroma/hematoma, one patient without a functioning shunt, and one due to perioperative intracranial bleeding) (Fig 1). Forty-three HI were evaluated, and one person was excluded because of an obvious gait disturbance. In total, 42 HI were included in the study.

Ninety-five percent of the patients reported symptoms of iNPH for more than one year and the most commonly occurring symptom at onset was gait disturbance (80.5%). The patients had significantly more cardiovascular diseases/risk factors than the HI (hypertension, hyperlipidemia, TIA/stroke, and ischemic heart disease) and a larger proportion were smokers compared to the HI. The occurrence of diabetes mellitus, polyneuropathy, spinal stenosis, and arthrosis of the knee and hip was at the same level in both groups. The HI were significantly younger than the patients (71.1 ± 5.9 vs 75.7 ± 5.4; p < 0.01) (Table 1).

**Table 1:** Descriptive characteristics for patients and healthy individuals.

Characteristics	Patients n = 43	Healthy individuals n = 42	p-value
Age (years)	75.9 ± 5.5	71.1 ± 5.9	<0.001^a^
Sex (n male/female, female %)	26/17 (39.5)	17/25 (59.5)	0.084^b^
Body height (cm)	171.5 ± 11.6 n = 41	171.8 ± 9.2	0.906^a^
MMSE (0–30 p)	25.4 ± 2.7	29.4 ± 0.9	<0.001^a^
10 MWT (s)	16.84 ± 7.77	7.92 ± 1.18	<0.001^a^
10 MWT (steps)	28.14 ± 12.51	15.05 ± 1.85	<0.001^a^
Smoking	9 (21.4) n = 42	1 (2.4)	<0.015^b^
Diabetes	9 (21.4) n = 42	4 (9.5)	0.227^b^
Hypertension	27 (64.3) n = 42	8 (19.0)	<0.001^b^
Hyperlipidemia	24 (57.1) n = 42	6 (14.3)	<0.001^b^
TIA/Stroke	15 (35.7) n = 42	0 (0) n = 41	<0.001^b^
Ischemic heart disease	10 (23.8) n = 42	1 (2.4)	<0.007^b^
Polyneuropathy	2 (4.8) n = 42	2 (4.8)	1^b^
Spinal stenosis	5 (11.9) n = 41	1 (2.4)	0.202^b^
Arthrosis knee/hip	7 (16.7) n = 42	9 (21.4)	0.782^b^

Data are presented as mean ± standard deviation or n (%).

^a^t-test,

^b^Fishers’s exact test. MMSE = Mini mental state examination, 10 MWT = 10-meter walk test, TIA = Transient ischemic attack. Significance level p ≤ 0.05.

### Data collection

**Fig 2:**
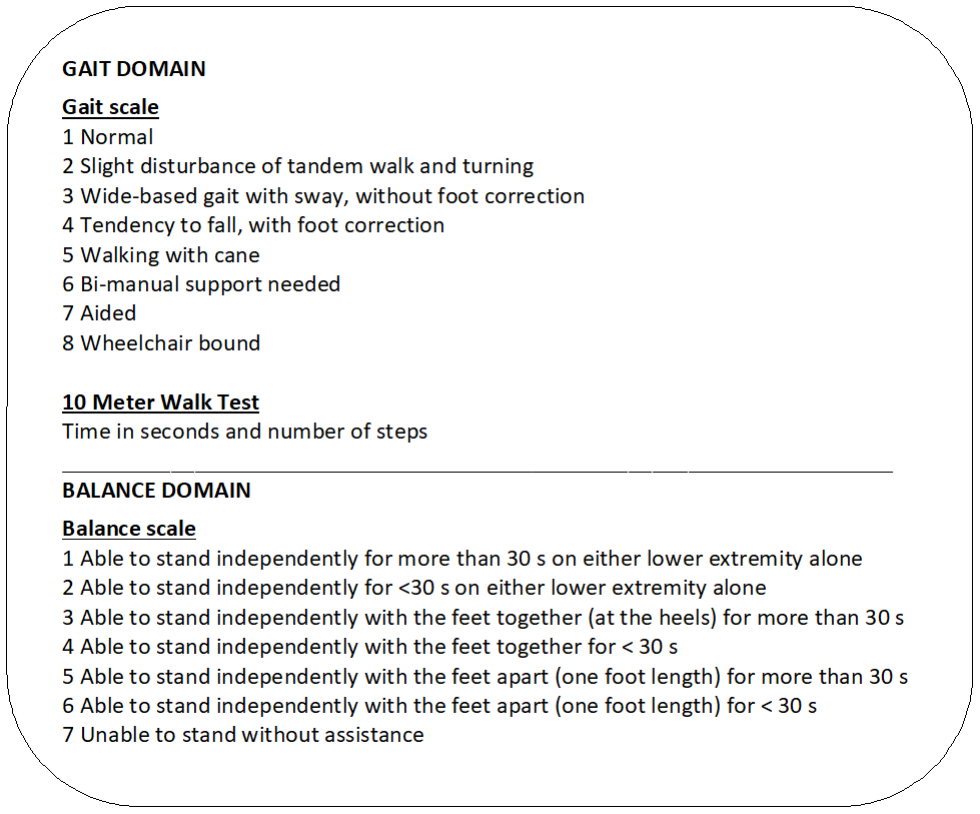
Instruments used in the iNPH scale gait domain and the balance domain. In the gait domain, the ordinal scale score and the results from the 10MWT are converted to a 0–100 score. In the balance domain, the ordinal scale score is converted to a 0–100 score [19].

The patients were examined preoperatively and three months postoperatively, while the HI were examined once. All included patients had a ventriculoperitoneal shunt insertion with a Codman Certas® Plus Valve. Cognitive assessment for the patients was conducted using the Mini Mental State Examination (MMSE) [22], and the neuropsychological tests included in the iNPH scale; Grooved pegboard, the Rey Auditory Verbal Learning Test and the Swedish Stroop test (19). The HI underwent the MMSE [22] examination. Functional mobility in both groups, measured in terms of time and number of steps, was evaluated using the 10MWT [20] and the TUG test [21] (TUG_time_, TUG_steps_). Additionally, the patients were assessed using an eight-graded ordinal gait scale included in the iNPH scale. The iNPH scale consists of four sub-scores converted to a 0–100 scale in the domains of neuropsychology, gait, balance, and continence. A total iNPH score is calculated based on all sub-scores [19]. In this study, the total iNPH scale score (iNPH scale_total_), the balance domain score (iNPH scale_balance_), (converted from an ordinal seven-graded scale) (Fig 2), and the gait domain score (iNPH scale_gait_), were used for correlation analyses. The gait domain score was calculated based on performance in the 10MWT (time and number of steps) and the grading from an ordinal gait scale [19] (Fig 2). The total iNPH scale score is the mean of the four sub-scores, with double weight given to the gait score (2x gait score + balance score + neuropsychology score + continence score) divided by 5 or the number of included scores [19].

**Fig 3:**
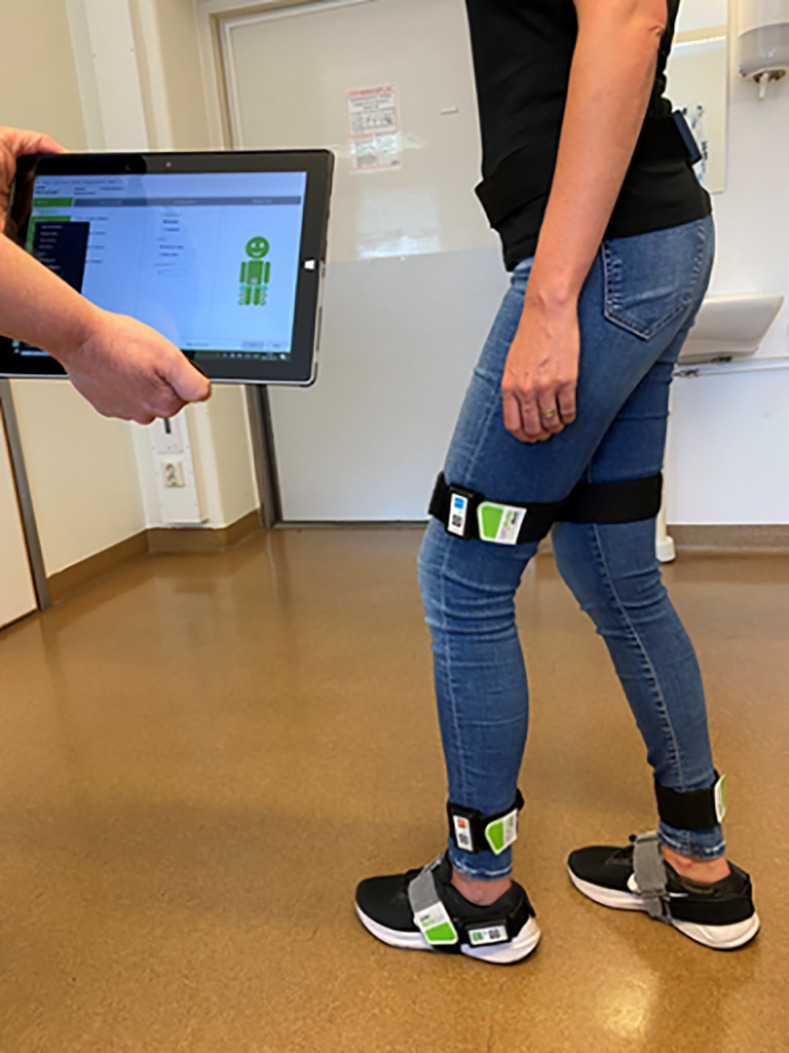
The RehaGait® system.

Quantitative gait parameters for both the patient group and the HI group were examined using the RehaGait® system (HASOMED, Magdeburg, Germany), (Fig 3). Motion data is collected using 3-axial accelerometers, gyroscopes, and magnetometers, and the data is transferred and stored in a tablet via a Bluetooth connection. The system processes inertial measurement data using a combination of models and assumptions to analyze gait movements. All values are based on gait events derived from inertial data collected by the foot sensors. The sensors attached to the foot, shank, thigh and hip calculate their orientation and set in relation to each joint. These values are combined with data from static and dynamic calibration movement to derive the joint angles in body planes. For velocity RehaGait® uses a Zero Velocity Update method that resets the velocity to zero during moments when the foot is stationary. Additionally, Kalman-like filters are used to estimate dynamic error and noise to compensate for drift. For position both foot sensors are set in relation to calculate a body center, that is used as boundary condition to reduce positional errors.

The system has been validated in healthy older adults, showing good to excellent reliability with intraclass correlation coefficient values ranging from 0.73 to 0.99 [23]. The research participants in the present study walked along a corridor, free from any disturbing activities, at a self-selected speed for approximately 20 meters, with the test leader (JR) beside the participant to ensure safety. Each session was performed twice, and the mean of each parameter was calculated. Three types of gait parameters were collected: basal parameters (velocity, cadence, stride length, stride duration, and variability), gait cycle parameters (stance, swing, double support, and single support) and joint kinematics (hip, knee, and foot joint angles and step height).

### Statistical analysis

Statistical analyses were conducted using SPSS Statistics Version 29 (IBM Corp, Armonk, NY, USA). A prior sample size estimation with a significance level of 5%. was conducted based on the mean difference of cadence between patients and HI in previous studies. With 80% power 26 participants were needed in each group. The normality of distributions was assessed using the Shapiro-Wilk’s test. Between-group differences in descriptive variables were examined using the t-test or Fisher’s exact test. Within-group differences were assessed using the t-test and 95% confidence interval (CI) or a Wilcoxon signed rank test and Hodges-Lehman median difference, depending on the normality of distributions. Because of the age differences between patients and HI, differences in the TUG test and gait variables between patients and the HI were assessed with analysis of covariance (ANCOVA) with adjustment for age and Bonferroni correction for multiple tests at the pre- and post-comparisons. If non-normally distributed data, the Quade nonparametric analysis of covariance with adjustment for mean age was used. Mean, standard error (SE), 95% CI and mean difference or median and interquartile range (IQR) were used in the presentation. Differences between gait cycle variables and joint kinematic variables in the left and right legs were examined. No significant differences were found for any variable, and therefore, the left and right variables were analyzed as a mean composite variable. Correlations between variables were calculated with the Spearman rank correlation analysis, with correlation coefficients interpreted as follows: a rho value of 0.90–1.00 signifying a very strong correlation, 0.70–0.89 indicating a high correlation, 0.40–0.69 representing a moderate correlation, 0.10–0.39 reflecting a weak correlation, and 0.00–0.09 indicating a negligible correlation [24]. The significance level for all tests was set at p ≤ 0.05.

## Results

Preoperatively, the patients performed the TUG test significantly worse than the HI, TUG_time_ (patients: mean 20.41; CI 18.08–22.74 s vs HI: mean 8.90; CI 6.54–11.26 s, p < 0.001), TUG_steps_ (patients: 28.81; CI 25.57–32.06 steps vs HI: 13.59; CI 10.30–16.87 steps, p < 0.001). Postoperatively, significant differences remained, TUG_time_ (patients 12.70; CI 11.75–13.64 s vs HI 8.67; CI 7.80–9.55 s, p < 0.001), TUG_steps_ (patients 18.46; CI 17.14–19.78 steps vs HI 13.19; CI 11.96–14.42 steps, p < 0.001).

The iNPH scale scores improved after shunt surgery, total score (mean difference 13.87; CI 10.58–17.16, p < 0.001), gait score (mean difference 20.87; CI 14.73–27.00, p < 0.001) and balance score (mean difference 6.05; CI 1.95–10.15, p = 0.005) (Table 2).

**Table 2:** Functional tests and qualitative gait variables in the patient group, pre- and postoperatively.

	Patients preoperatively (n = 43)	Patients postoperatively (n = 36)		
	Mean ± SD or Median (IQR)	Mean ± SD or Median (IQR)	Mean difference 95% CI	p-value
**Functional tests**				
TUG (s)	20.75 ± 10.23	12.84 ± 3.75	6.51(4.98–8.95)	<0.001^a^
TUG (steps)	29.41 ± 14.33	18.70 ± 5.37	8.41(5.59–11.22)	<0.001^a^
iNPH scale total	53.40 ± 12.10	68.40 ± 15.46	13.87(10.58–17.16)	<0.001^a^
iNPH scale gait	43.67 ± 19.09	66.13 ± 23.76	20.87(14.73–27.00)	<0.001^a^
iNPH scale balance	69.95 ± 11.00	75.97 ± 13.07	6.05(1.95–10.15)	0.005^a^
**Basal gait parameters**				
Stride length (m)	0.79 ± 0.27	1.05 ± 0.25	0.22(0.16–0.28)	<0.001^a^
Stride duration (s)	1.18 ± 0.12	1.13 ± 0.11	0.06(0.02–0.10)	0.005^a^
Velocity (m/s)	0.68 ± 0.23	0.93 ± 0.25	0.22(0.15–0.29)	<0.001^a^
Cadence (steps/min)	103.16 ± 10.85	107.45 ± 9.76	4.83(1.48–8.17)	<0.001^a^
Variability (time)	4.10(3.54–7.21)	4.06(3.09–5.21)	0.97(0.28–1.97)^c^	0.007^b^
Variability (spatial)	7.84(6.00–11.97)	8.22(6.41–10.83)	0.62(0.70–2.08)^c^	0.318^b^
**Gait cycle parameters**				
Stance (%)	66.55 ± 2.94	65.36 ± 2.56	0.96(0.09–1.83)	<0.001^a^
Swing (%)	33.47 ± 2.91	34.29 ± 3.03	0.70(−0.38–1.78)	0.194^a^
Single support (%)	33.59 ± 2.58	34.77 ± 2.29	1.17(0.34–1.99)	<0.001^a^
Double support (%)	16.47 ± 2.79	15.21 ± 2.31	1.18(0.36–2.00)	<0.001^a^
**Joint kinematics**				
Heel strike (°)	12.44 ± 6.91	18.69 ± 7.34	6.12(3.97–8.27)	<0.001^a^
Toe-off (°)	36.87 ± 10.75	47.51 ± 9.93	9.42(6.65–12.20)	<0.001^a^
Knee extension (°)	0.45 ± 0.05	0.45 ± 0.09	0(−0.03–0.04)	0.780^a^
Knee flexion (°)	37.74 ± 11.84	43.05 ± 8.16	5.68(3.37–8.00)	<0.001^a^
Hip extension (°)	4.84 ± 3.22	5.62 ± 3.85	0.41(−1.93–1.12)	0.589 ^a^
Hip flexion (°)	21.09 ± 7.60	29.61 ± 5.29	7.80(4.95–10.65)	<0.001^a^
Step height (cm)	12.20 ± 3.00	13.95 ± 2.14	1.75(0.97–2.53)	<0.001^a^

SD = standard deviation, IQR = interquartile range, CI = confidence interval.

^a^t-test,

^b^Wilcoxon signed rank test,

^c^Hodges-Lehman median difference. Significance level p ≤ 0.05.

Variability time (step to step fluctuation related to the stride duration), variability spatial (step to step fluctuation related to the stride length).

### Basal gait parameters

The patients exhibited significantly shorter stride length compared to the HI group (mean difference 0.50; CI 0.40–0.60 m, p < 0.001) and reduced gait velocity (mean difference 0.54; CI 0.45–0.63 m/s, p < 0.001) before the shunt surgery. The patients also demonstrated poorer performance in stride duration (mean difference 0.10; CI 0.05–0.15 s, p < 0.001), cadence (mean difference 9.38; CI 4.87–13.88 steps/min, p < 0.001), and variability time (p < 0.001) and spatial (p < 0.001) (Table 3). Following the shunt surgery, the patients showed significant improvements in all basal gait parameters, except for spatial variability (p = 0.318). The largest improvements were observed in stride length (mean difference 0.22; CI 0.16–0.28 cm, p < 0.001) and velocity (mean difference 0.22; CI 0.15–0.29 m/s, p < 0.001) (Table 2). Despite these improvements, significant differences between the patients and HI persisted in all basal gait parameters after the surgery, particularly in stride length (mean difference 0.24; CI 0.15–0.33, p < 0.001) and velocity (mean difference 0.28; CI 0.18–0.37, p < 0.001) (Table 3).

**Table 3:** Quantitative gait variables, comparisons between the patients and the healthy individuals.

	Patients (n = 43)		Healthy individuals (n = 42)			
	Mean	95% CI	Mean	95% CI	Mean difference 95% CI	p-value
Stride length (m) Pre	0.81	0.74–0.88	1.31	1.24–1.38	0.50 (0.40–0.60)	<0.001^a^
Post	1.07	1.00–1.13	1.31	1.25–1.37	0.24 (0.15–0.33)	<0.001^a^
Stride duration (s) Pre	1.17	1.14–1.20	1.07	1.04–1.10	0.10 (0.05–0.15)	<0.001^a^
Post	1.12	1.01–1.16	1.07	1.04–1.10	0.05 (0.01–0.10)	0.028^a^
Velocity (m/s) Pre	0.70	0.64–0.76	1.23	1.17–1.30	0.54 (0.45–0.63)	<0.001^a^
Post	0.96	0.89–1.03	1.23	1.17–1.30	0.28 (0.18–0.37)	<0.001^a^
Cadence (steps/min) Pre	103.70	100.66–106.74	113.08	110.00–116.16	9.38 (4.87–13.88)	<0.001^a^
Post	108.02	104.88–111.17	113.16	110.31–116.01	5.13 (0.72–9.55)	0.023^a^
Variability (time) Pre	4.10 (3.54–7.21)^b^		3.17 (2.70–3.64)^b^			<0.001^c^
Post	4.06 (3.09–5.21)^b^		3.17 (2.70–3.64)^b^			0.025^c^
Variability (spatial) Pre	7.84 (6.00–11.97)^b^		3.80 (3.39–4.72)^b^			<0.00^c^
Post	8.22 (6.41–10.83)^b^		3.80 (3.39–4.72)^b^			<0.001^c^
Stance (%) Pre	66.53	65.78–67.27	63.61	62.87–64.34	2.92 (1.83–4.01)	<0.001^a^
Post	65.31	64.59–66.03	63.62	62.98–64.27	1.68 (0−68–2.69)	<0.001^a^
Swing (%) Pre	33.48	32.75–34.21	36.45	35.73–37.18	2.97 (1.89–4.04)	<0.001^a^
Post	34.30	33.47–35.13	36.46	35.73–37.18	2.16 (1.01–3.30)	<0.001^a^
Single support (%) Pre	33.63	32.97–34.28	36.55	35.89–37.21	2.92 (1.96–3.89)	<0.001^a^
Post	34.80	34.15–35.44	36.56	35.97–37.15	1.77 (0.85–2.68)	<0.001^a^
Double support (%) Pre	16.43	15.71–17.14	13.63	12.93–14.34	2.79 (1.74–3.84)	<0.001^a^
Post	15.17	14.52–15.83	13.63	13.03–14.23	1.54 (0.62–2.46)	<0.001^a^
Heel strike (°) Pre	12.73	10.96–14.51	28.19	26.41–29.96	15.45 (12.84–18.06)	<0.001^a^
Post	19.39	17.45–21.33	27.89	26.13–29.65	8.50 (5.78–11.23)	<0.001^a^
Toe-off (°) Pre	37.27	34.39–40.15	59.69	56.85–62.53	22.44 (18.21–26.64)	<0.001^a^
Post	48.70	45.92–51.47	60.00	56.59–61.61	10.40 (6.51–14.30)	<0.001^a^
Knee extension (°) Pre	0.45	0.44–0.46	0.50	0.49–0.51	0.05 (0.03–0.07)	<0.001^a^
Post	0.46	0.43–0.48	0.50	0.48–0.52	0.04 (0.01–0.07)	0.014^a^
Knee flexion (°) Pre	38.05	35.01–41.1	50.27	47.14–53.40	12.21 (7.68–16.75)	<0.001^a^
Post	43.66	41.09–46.23	50.09	47.77–52.40	6.43 (2.83–10.02)	<0.001^a^
Hip extension (°) Pre	5.26	4.23–6.30	8.66	7.67–9.66	3.40 (1.89–4.91)	<0.001^a^
Post	6.00	4.78–7.22	8.75	7.66–9.84	2.75 (1.05–4.46)	0.002^a^
Hip flexion (°) Pre	20.67	18.78–22.56	36.09	34.18–38.01	15.42 (12.64–18.21)	<0.001^a^
Post	29.91	28.30–31.51	35.43	34.00–36.86	5.52 (3.30–7.74)	<0.001^a^
Step height (cm) Pre	12.20	11.30–13.10	14.50	13.60–15.30	2.30 (1.00–3.60)	<0.001^a^
Post	14.10	13.30–14.90	14.4	13.60–15.10	0 (−0.01–0.01)	0.641^a^

CI = Confidence interval, Pre = Preoperatively, Post = Postoperatively.

^a^Analysis of covariance with adjustment for age and Bonferroni correction for multiple tests. Means are presented in adjusted values. Patients were evaluated pre- and postoperatively and were compared the healthy individuals evaluated once. The means are adjusted at both comparisons.

^b^Median (IQR) in unadjusted values,

^c^Quade nonparametric analysis of covariance with adjustment for mean age. Significance level p ≤ 0.05. Variability time (step to step fluctuation related to the stride duration), variability spatial (step to step fluctuation related to the stride length).

### Gait cycle parameters

Preoperatively, the patients exhibited significantly longer stance phase and shorter swing phase compared to the HI group (stance: patients 66.53; CI 65.78–67.27% vs HI 63.61; CI 62.87–64.34%; p > 0.001, swing: patients 33.48; CI 32.75–34.21% vs HI 36.45; CI 35.73–37.18%, p > 0.001). Similar differences were observed in single support (patients 33.63; CI 32.97–34.28% vs HI 36.55; CI 35.89–37.21%, p > 0.001) and double support (patients 16.43; Ci 15.71–17.14% vs HI 13.63; CI 12.93–14.34%; p < 0.001) (Table 3). The shunt surgery had no significant effect on the patients’ swing phase (p = 0.194), but there were small yet significant improvements in stance (mean difference 0.96; CI 0.09–1.83%, p < 0.001), single support (mean difference 1.17; CI 0.34–1.99%, p < 0.001), and double support (mean difference 1.18; 0.36–2.00%, p < 0.001) (Table 2). After the surgery, significant differences between the patients and the HI group persisted in all gait cycle parameters (Table 3).

### Joint kinematics

All preoperative joint kinematic variables were significantly worse in the patient group and the most significant differences between the patients and the HI group were observed in heel strike angle (mean difference 15.45; CI 12.84–18.06°, p < 0.001), toe-off angle (mean difference 22.44; CI 18.21–26.64°, p < 0.001), hip flexion (mean difference 15.42; CI 12.64–18.21°, p < 0.001) and knee flexion (mean difference 12.21; CI 7.68–16.75°, p < 0.001). Both the patients and HI group exhibited very small angles in knee extension (Table 3). The shunt surgery had significant effect on step height in the patient group (mean difference 1.75; CI 0.97–2.53 cm, p < 0.001) (Table 2), and the significant difference between the patients and HI group disappeared after surgery (p = 0.641) (Table 3). Shunt surgery had significant effects on heel strike angle (p < 0.001), toe-off angle (p < 0.001), knee flexion (p < 0.001), and hip flexion (p < 0.001) (Table 2). However, significant differences between the patients and the HI group persisted after surgery, particularly in heel strike angle (mean difference 8.50; CI 5.78–11.23°, p < 0.001), toe-off angle (mean difference 10.40; CI 6.51–14.30°, p < 0.001), hip flexion (mean difference 5.52; CI 3.30–7.74, p < 0.001), and knee flexion (mean difference 6.43; CI 2.83–10.02, p < 0.001) (Table 3).

### Correlations between quantitative gait parameters and clinical tests preoperatively

The strongest correlations between the clinical tests measuring functional mobility (TUG_time_ and TUG_steps_) and the iNPH scale scores, and the quantitative gait parameters, were observed among the basal gait parameters. Stride length and velocity showed strong negative correlations with TUG_time_, TUG_steps_ (rho ≤ −0.79) and strong correlations with iNPH scale_total_, and iNPH scale_gait_ (rho ≥ 0.83). Heel strike angle in joint kinematics exhibited strong negative correlations with TUG_steps_ (rho = −0.75) and strong correlation with INPH scale_gait_ (rho = 0.77), while toe-off angle showed strong negative correlations with TUG_time_ (rho = −0.71), TUG_steps_ (−0.76), and strong correlation with INPH scale_gait_ (0.80). Knee flexion demonstrated a strong correlation with iNPH scale_total_ (rho = 0.72). All gait cycle parameters showed moderate negative/positive correlations with TUG_time_, TUG_steps_, and the iNPH scale scores, except for the iNPH scale_balance_ (rho −0.12–0.12). The correlation between iNPH scale_balance_ and all quantitative gait parameters was weak or negligible (Table 4).

**Table 4:** Correlations between quantitative gait parameters and functional mobility and iNPH scale scores in the patient group preoperatively.

**Basal gait parameters**	Stride length	Stride duration	Velocity	Cadence	Variability time	Variability spatial	
TUG (time)	−0.79	0.30	−0.87	−0.32	0.50	0.60	
TUG (steps)	−0.87	0.02	−0.85	−0.02	0.42	0.67	
iNPH scale Total	0.83	−0.09	0.84	0.07	−0.56	−0.66	
iNPH scale Gait	0.88	−0.16	0.88	0.13	−0.57	−0.69	
iNPH scale Balance	0.25	−0.14	0.27	0.06	−0.33	−0.39	
**Gait cycle parameters**	Stance	Swing	Single support	Double support			
TUG (time)	0.53	−0.51	−0.53	0.53			
TUG (steps)	0.50	−0.50	−0.51	0.52			
iNPH scale Total	−0.65	0.66	0.65	−0.69			
iNPH scale Gait	−0.51	0.52	0.53	−0.55			
iNPH scale Balance	−0.10	0.12	0.09	−0.12			
**Joint kinematics**	Heel strike angle	Toe off angle	Knee extension	Knee flexion	Hip extension	Hip flexion	Step height
TUG (time)	−0.60	−0.71	−0.20	−0.62	−0.38	−0.57	−0.60
TUG (steps)	−0.75	−0.76	−0.17	−0.69	−0.39	−0.57	−0.67
iNPH scale Total	0.62	0.67	0.05	0.72	0.45	0.65	0.63
iNPH scale Gait	0.77	0.80	0.16	0.69	0.41	0.62	0.64
iNPH scale Balance	0.19	0.10	−0.17	0.20	−0.01	0.20	0.09
Spearman Rho Interpretations	0.00–0.09	0.10–0.39	0.40–0.69	0.70–0.89	0.91–1.00		
	negligible	weak	moderate	high	very strong		
	negligible	weak	moderate	strong	very strong		

Correlations were analyzed with Spearman Rho. Negative and positive correlations at the same level are coded with the same color. TUG = Timed Up and Go test, TUG time (sec), TUG steps (n), iNPH scale total, gait and balance (scores 0–100), stride length (m), stride duration (s), velocity (m/s), gait cycle parameters (%), joint kinematic angels (°), step height (cm).

## Discussion

The primary aim was to characterize gait in iNPH in comparison to HI. There are three basic approaches when characterizing gait [25], in this study defined as basal gait parameters, gait cycle parameters and joint kinematics. The novel contribution of this study lies in providing a detailed depiction of gait kinematics in iNPH. Patients with iNPH exhibited significant deviations in heel strike angle, toe-off angle, and hip flexion when compared to the HI group. These parameters showed improvement after shunt surgery but remained significantly worse compared to the HI group in the postoperative period. Notably, low step height is often considered a characteristic sign of iNPH [1,10]. In the present study, step height, measured as the maximum distance from the foot-attached sensor to the ground, was moderately affected preoperatively, and there were no differences between patients and controls postoperatively in this parameter, while other gait angles remained affected. The strong correlations observed between heel strike angle and toe-off angle and the clinical tests, the TUG test, and the iNPH_gait_ score, underscore the influence of ankle kinematics on gait speed and step length. These findings highlight the need for more detailed explanations of joint kinematics in the lower extremities when describing the gait pattern in iNPH.

The basal gait parameters are more commonly described in previous research. This study confirms previous results emphasizing that patients with iNPH exhibit slower walking speed, shorter strides [11–14,16,17,26], decreased cadence [11,12,16,17,26] and increased stride variability [11–14] compared to healthy individuals. In the present study, patients walked slightly faster before shunt surgery (0.68 ± 0.23 m/s) compared to the findings in previous research from Lim et al. (0.55 ± 0.05 m/s) [13] and He et al. (0.46 ± 0.18 m/s) [17]. Additionally, patients in the present study walked with longer strides (0.79 ± 0.27 m), than those reported in the aforementioned studies, 0.63 ± 0.05 m [13], 0.56 ± 0.22 m [17]. The variation in walking distance with Lim et al. covering 5.8 m [13] and He et al. covering 7 m [17], compared to the 20-meter walk performed in our study, could account for these differences. The actual walked distance may have an impact on gait speed in short-distance walking tests [27].

The gait cycle parameters differed significantly between the patient group and the HI group, both before and after surgery. The effects from shunt surgery on the proportion of time in stance, single support and double support were small and the proportion of time in the swing phase did not show any improvement after shunt surgery. The gait pattern, with increased proportion of time in stance and double support compared to the HI, remained after shunt surgery. In addition, the spatial variability (variation in stride length), did not improve after shunt surgery. Previous research has suggested that patients with iNPH have a conscious motor control component to establish dynamic stability, especially patients with a high fall risk [28]. These compensation strategies may be one of the remaining impairments in the gait cycle variables. INPH is defined as a higher-level gait disorder, with pathologies in the cortex and the basal ganglia, causing impaired locomotion and disequilibrium [29]. The pathophysiology behind the condition is of course the most pronounced factor explaining the gait pattern in iNPH. Also behavior patterns emerge, where for example cautious gait is an adaptation to impaired balance [28,29]. INPH patients have often had symptoms for a long time before surgery [3], with long time for adaptation. In the present study, a shunt insertion had a large effect on stride length and velocity, heel strike angle and toe-off angle, but all these parameters were still significantly worse compared to the HI. The differences between the groups remained on stride length, velocity, heel strike angle and hip flexion. Walking speed is a determinant of cadence and stride length, and joint movements increase with increasing gait speed in older adults [30]. With specific rehabilitation strategies, walking speed, and related gait parameters may improve further after shunt surgery. The results from a previous actigraphic study from our research unit showed that iNPH patients did not increase their daily activity level after shunt surgery, despite improved functions on clinical tests [31]. Analyses of the behavior patterns and understanding of comorbidities are important in the planning of the rehabilitation among iNPH patients.

The strong correlations between the iNPH scale_gait_ (including the 10 MWT time and steps), the TUG_time_ and the ankle joint angles, reflect the correlation between gait speed and joint kinematics [30]_._ The corresponding correlations are seen in the number of steps in the TUG_steps_ and the heel strike angle and the toe-off angle. Longer strides generate greater joint angles, especially in the ankle joint, but are also seen in the knee flexion, hip extension and hip flexion. Preoperatively, the patients walked with reduced step height. The step height was the only parameter that was normalized postoperatively even with the remaining reduction of heel strike angle and toe-off angle. This result highlights the importance of analyzing the joint kinematics in more detail; step height does not explain the complexity of the ankle joint motion in iNPH. Patients landed flat-footed with reduced ankle joint angles in the present study. Rolling through the step and generating a good roll-off is essential to induce force in the forward progression [25]. In the present study no analyses were performed to determine whether walking speed is a predictor of other parameters and if significant differences remain if controlled for walking speed. This is an important field to analyze in future research.

There were no correlations for any gait parameter and the iNPH scale balance domain score. Dynamic gait stability in iNPH has previously been described and the influence of voluntary cautiousness has been discussed [32,33]. Nikaido et al. reported that patients with iNPH walked with poorer lateral postural control which may be caused by the iNPH symptoms and with poorer anterior-posterior control, which may not be a factor related to only gait and balance disturbance but also due to a voluntary cautious strategy [32]. Dynamic postural control is probably an important factor in the gait disturbance in iNPH, and more research is needed to explore different aspects of dynamic balance and their influences in gait [34]. Static postural control measured with the iNPH scale cannot be transferred to understand the gait pattern in iNPH due to the low sensitivity in the ordinal balance scale.

In most of the gait variables, the standard deviation in the patient group was approximately twice as large as in the HI group, reflecting the wide range of variation of gait symptoms among iNPH patients. One aspect for future research is to analyze if there are differences in quantitative gait outcomes after shunt surgery depending on symptom severity. Previous research has indicated that a longer waiting time for surgery has a negative influence on postoperative outcomes in iNPH [35,36].

### Strengths and limitations

The patients were consecutively included through an extensive evaluation process at the University Hospital in Linköping. It is a strength of this study that the evaluation followed a strict protocol and patients with suspected iNPH in the south-east part of Sweden were evaluated in the same standardized way. Surgery was performed by three highly experienced surgeons and the patients were carefully evaluated with MRI before the postoperative assessments, to control for complications. However, it is a limitation that 13 individuals had to be excluded because of increased symptoms and subacute surgery. These patients were not available for the gait analysis. In addition, this study only reflects patients walking without walking aids. This exclusion criterion was used in order to analyze the normal walking pattern without influences from walking aids. However, this choice of method entailed the exclusion of patients with the most severe gait symptoms from the analysis. These factors may lead to selection bias affecting the ability to generalize the results to the general iNPH population.

The conclusion and the ability to reproduce the study may be affected by some study design biases. The design with repeated measures pre- and postoperatively entails risk of regression to the mean effects. To reduce the effect the patients were evaluated twice at baseline and twice postoperatively but without time span. We also used a control group. The control group was only assessed on one occasion which is a limitation because the pre- and postoperative assessments were compared with the same HI assessment. However, the probability of a change in the HIs gait performance over a period of three months is low. The HI were recruited by an advertisement and not randomly recruited. Additionally, the recruited HI were very active and especially interested in research, and they may not have reflected a randomized group of elderly.

An inertial sensor system is portable and has a relatively low cost compared to fixed optical motion capture systems or floor motion systems in specialized labs. A system with wearable sensors entails also a more real-life situation when walking in a corridor. RehaGait® was easy to use in our clinical practice and the calibration and use were easy to teach. Two sensors broke during the evaluation period and six participants could not be evaluated during the repair time. However, there were limitations because the software in the RehaGait® system reports selected variables. There was no access to raw inertial measurement unit data and no ability to derive other parameters. The RehaGait® is validated for elderly [23] but not for patients with iNPH, and for example broad-based gait, often emphasized in iNPH, could not be measured. Additionally, the sensors are attached to the lower body which excludes analysis of the arm motions.

## Conclusion

The iNPH patients walked with reduced hip flexion, heel strike angle and toe-off angle together with shorter strides, decreased velocity and increased time for swing and single support, compared to HI. Step height improved to normal after shunt surgery, and the other gait characteristics improved to some extent but remained significantly worse compared to the HI group. Heel strike angle and toe-off angle had strong correlations with the iNPH scale gait domain and TUG test, indicating the influence of ankle kinematics on gait speed and step length. More research is needed about how walking speed affects joint kinematics in iNPH.

## Supporting information

S1 Dataset Dataset of the analyzed data. (XLSX)
